# Continuous and early prediction of future moderate and severe Acute Kidney Injury in critically ill patients: Development and multi-centric, multi-national external validation of a machine-learning model

**DOI:** 10.1371/journal.pone.0287398

**Published:** 2023-07-25

**Authors:** Francesca Alfieri, Andrea Ancona, Giovanni Tripepi, Andrea Rubeis, Niccolò Arjoldi, Stefano Finazzi, Valentina Cauda, Riccardo Maria Fagugli

**Affiliations:** 1 U-Care Medical srl, Torino, Italy; 2 CNR-IFC, Clinical Epidemiology and Pathophysiology of Renal Diseases and Hypertension, Reggio Calabria, Italy; 3 Department of Applied Science and Technology, Politecnico di Torino, Turin, Italy; 4 Dipartimento di Salute Pubblica, Laboratorio di Clinical Data Science, Istituto di Ricerche Farmacologiche Mario Negri IRCCS, Ranica, Bergamo, Italy; 5 S.C. Nefrologia e Dialisi, Azienda Ospedaliera di Terni, Terni, Italy; CHU Nantes, FRANCE

## Abstract

**Background:**

Acute Kidney Injury (AKI) is a major complication in patients admitted to Intensive Care Units (ICU), causing both clinical and economic burden on the healthcare system. This study develops a novel machine-learning (ML) model to predict, with several hours in advance, the AKI episodes of stage 2 and 3 (according to KDIGO definition) acquired in ICU.

**Methods:**

A total of 16’760 ICU adult patients from 145 different ICU centers and 3 different countries (US, Netherland, Italy) are retrospectively enrolled for the study. Every hour the model continuously analyzes the routinely-collected clinical data to generate a new probability of developing AKI stage 2 and 3, according to KDIGO definition, during the ICU stay.

**Results:**

The predictive model obtains an auROC of 0.884 for AKI (stage 2/3 KDIGO) prediction, when evaluated on the internal test set composed by 1’749 ICU stays from US and EU centers. When externally tested on a multi-centric US dataset of 6’985 ICU stays and multi-centric Italian dataset of 1’025 ICU stays, the model achieves an auROC of 0.877 and of 0.911, respectively. In all datasets, the time between model prediction and AKI (stage 2/3 KDIGO) onset is at least of 14 hours after the first day of ICU hospitalization.

**Conclusions:**

In this study, a novel ML model for continuous and early AKI (stage 2/3 KDIGO) prediction is successfully developed, leveraging only routinely-available data. It continuously predicts AKI episodes during ICU stay, at least 14 hours in advance when the AKI episode happens after the first 24 hours of ICU admission. Its performances are validated in an extensive, multi-national and multi-centric cohort of ICU adult patients. This ML model overcomes the main limitations of currently available predictive models. The benefits of its real-world implementation enable an early proactive clinical management and the prevention of AKI episodes in ICU patients. Furthermore, the software could be directly integrated with IT system of the ICU.

## Introduction

Acute Kidney Injury (AKI) is a sudden loss of the kidney function and represents a worldwide concern due to the high in-hospital incidence, mortality, social costs and disability [1, 2]. The early prediction of AKI could allow timely intervention in diagnosis and treatment, with the aim to facilitate reversible forms of AKI [3]. The clinical approach adopted by physicians to limit the AKI onset in Intensive Care Units (ICU) consists mainly in the prevention of the disease, by adopting measures able to guarantee appropriate volume control, kidney perfusion, sepsis prevention and nephrotoxic drug tailoring. Several researches developed algorithms by the use of big data analysis and Artificial Intelligence (AI) instruments, but until now the presented studies are heterogeneous and many of them are not externally validated or contain several biases [4]. We have previously conducted a study on patients who developed AKI stage 2 and 3 within the ICU, according to KDIGO, and classified them on the basis of the sole urine output [5]. That study analysed an international dataset by the use of Artificial Intelligence with the Deep Learning method, with the aim to identify a predictive algorithm for oliguric AKI. We observed that urine output trends can predict with accuracy the event at least with 12 hours in advance. An external validation of this study was also performed, and the validity and generalization of the approach were confirmed [6]. Due to the fact that oliguric AKI represents about 40–50% of the whole cases [7–9], in the present work we aim to extend the analysis to all forms of AKI. In particular, given the predictive purpose of our model, we focus on hospital-acquired-AKI Stage 2 and 3 of KDIGO classification, which is known from the literature to be approximately 50% of the total AKI episodes registered within ICU hospitalization [10–12].

This retrospective study is based on four different databases obtained from single-center hospitals, the MIMIC-III [13] and the AmsterdamUMC databases [14], and from multi-centre databases, the eICU [15] and Margherita Tre [16] ones, with the aim to test the generalized prediction capability of the model in different patient subpopulations and geographies.

## Materials and methods

### Data sources

The data used to generate a final database for the study originate from four different sources:

- MIMIC-III [13], a single-center database from patients admitted at the Beth Israel Deaconess Medical Center in Boston (MA) from 2001 to 2012 (53’423 admissions, 38’597 distinct patients);
- eICU collaborative research multicenter database [15], which contains data from more than 200 different United States ICUs, registered from 2014 to 2015 (200’859 admissions, 139’367 patients);
- AmsterdamUMC database [14], which contains anonymized data of 20’109 European patients admitted to the University hospital ICU of Amsterdam, in the Netherlands between 2003 and 2016, for a total of 23’106 admissions;
- Margherita Tre database, developed by the Group for the Evaluation of Interventions in Intensive Care Medicine (GiViTI) [16, 17] and available for research purposes, which contains data from 27 different Italian centers (60’430 ICU admissions, 55’702 patients) acquired from 2001 to 2022.

### Ethics approval and consent to participate

The study was conducted according to the Declaration of Helsinki.

The protocol of data collection for the Margherita Tre dataset was reviewed by the coordinating ethics committee, Comitato Etico Indipendente di Area Vasta Emilia Centro (CE-VAC), and by each local ethics committee of the hospitals adopting the MargheritaTre software. The informed consent was collected in agreement with national regulations.

The remaining datasets were fully deidentified before access. Deidentified data constitute nonhuman subject research, thus no institutional or ethical approvals were required for this study. Participant informed consent was not required for these datasets.

MIMIC-III project was evaluated and approved by the Massachusetts Institute of Technology (No. 0403000206) and the Institutional Review Board of the Beth Israel Deaconess Medical Center (2001–P–001699/14). Individual patient consent was avoidable since all sensible data and health information were deidentified, also, the project did not impact clinical care.

The eICU was not assessed by an institutional review board because of its retrospective nature, absence of direct patient intervention, and the inability of patient re–identification in compliance with the safety standards by Privacert (Cambridge, MA) (Health Insurance Portability and Accountability Act Certification no. 1031219–2).

The privacy audit on AmsterdamUMCdb has concluded that reidentification of patients from this database is not reasonably likely, and it can therefore be considered as anonymous information, in the context of the U.S. Health Insurance Portability and Accountability Act, the European General Data Protection Regulation (GDPR) and Dutch national Laws. The ethics audit has also concluded that responsible data sharing imposes minimal burden, whereas the potential benefit is tremendous.

### Inclusion and exclusion criteria

Male and Female patients older than 18 years of age and admitted to any intensive care units have been considered for the study. Re-admissions to the ICU were also included. Patients who had a stage 2 or 3 KDIGO AKI diagnosis at ICU admission, or initiated a renal replacement treatment, within the first 12 hours of ICU stay were excluded. Missing values of relevant clinical data for more than 4 consecutive hours as it is the case of urine output, heart rate and mean arterial pressure (MAP), or missing laboratory measurements for more than 4 days indicates exclusion criteria (Table 1).

**Table 1:** Exclusion criteria for the multi-centre retrospective study of patients admitted to ICUs.

Exclusion Criteria
• Age < 18 years • sCr baseline < 0.5 mg/dL
• Patients with AKI (stage 2/3 KDIGO) diagnosis or in renal replacement treatment within the first 12 hours of ICU stay • Patients with missing values for more than 4 consecutive hours of urine output and heart rate and MAP data more than 4 days of laboratory measurements data

### Definition

AKI: defined on the basis of Acute Kidney Injury KDIGO guidelines (2012) [18].

Features: represent the variables used as input parameters in mathematical models.

### Software specifications

Statistical analyses, extract-transform-load (ETL) process and data processing were performed using Python (version 3.7.9; Python Software Foundation: http://www.python.org). The Random Forest model was implemented with scikit-learn library in Python.

### Imputation of missing values

Values of features out of the permitted range were removed before starting the imputation phase [19].

Since parameter measurements were reported irregularly during patient ICU stay, the reconstruction of time series with the same sampling frequency (equals to 1 hour) was required for model development and validation.

Missing values were imputed with a forward-filling methodology using the last observed value. For the laboratory measurements, data were imputed for a maximum of 4 days [19], while hourly Urine Output, heart rate and mean arterial pressure values for a maximum of 4 hours.

The original average acquisition frequency of parameters in the different datasets is shown in Supplementary Info A, S1 Table in S1 File. In brief, haematological and biochemical measurements are generally collected every 12–24 hours, while urine output showed a sampling frequency of approximately 1–1.5 hours in all datasets and heart rate sampling frequency varies within 0.5–1 hours.

Urine Output values were normalized by the adjusted body weight of the patient, whose calculation includes additional parameters like height, weight at the admissions, and gender [20].


IdealBodyweight(IBW)(m)=50+(0.91×[h−152.4])

IdealBodyweight(IBW)(f)=45.5+(0.91×[h−152.4])

(m=male,f=female,h=heightincentimeters)

Adjustedbodyweight(adjBW)=IBW+0.4*(ABW–IBW)

(ABW=actualbodyweightattheICUadmission)

AKI criteria, as KDIGO classification, require the baseline value of Serum Creatinine (bSCr). Various methods for determining the baseline value of Serum Creatinine (sCr) values have been described and there is not a universally accepted single method. Due to the absence of pre-ICU admission sCr measurements for most ICU patients, we used the nadir (minimum) in-hospital value of bSCr.

### Sample labelling and model training

We considered the task of predicting AKI episodes of stage 2 and 3 KDIGO developed during an ICU stay as a classification problem. As classification labels, we assigned a “positive” label at each patient who experienced an AKI event. The portion of stay considered in the analysis was truncated at the onset of AKI. Patients who did not develop AKI were labeled as “negative”. Their ICU-stay were entirely considered for analysis.

The analysis was conducted by using a Random Forest classifier with the final objective of predicting the onset of AKI (stage 2/3 KDIGO) acquired in ICU. An additional model was used as a “baseline model” and described in Supplementary Info B: Logistic Regression model in S1 File.

The risk probability of developing AKI (stage 2/3 KDIGO) in the following hours is returned by the model. This output is represented as a score, it is updated every hour during the ICU stay.

We used ICU admissions equal to 60% of the total MIMIC-III and AmsterdamUMC population to train the models.

### Feature selection

To develop our model for the early prediction of risk to develop AKI (stage 2/3 KDIGO), a set of routinely collected hematological and biochemical measurements were extracted and processed to derive input features. The goal of the feature selection process was to obtain a minimum set of predictive and routinely-collected parameters to enable real-world integration of the model in clinical practice. We started the analysis by considering a series of clinical, anamnestic, and demographic data, generally believed to have a role in AKI development. Subsequently, a feature selection was conducted based on the model’s feature importance to identify a narrow set of more predictive parameters. The impurity-based feature importance, or Gini importance [21], was computed as the reduction of the criterion brought by that feature. According to their contribution in improving classification performance, all clinical parameters were inserted one by one as input features, and the resulting area under Receiving Operator Curve (auROC) was used to detect the minimum set of clinical parameters useful to enhance model’s performance.

### Model evaluation

Model coefficients obtained from the training set were fixed and applied to patients in the test sets as though they were observed prospectively. Specifically, for each patient in the test set, as new data became available, the predictive score was recomputed every hour. This resulted in hourly-updated risk score for AKI (stage 2/3 KDIGO) development for each patient.

As a primary evaluation metric, auROC was considered in predicting AKI (stage 2/3 KDIGO). The ROC curve and the auROC were obtained by varying the threshold that determined which patients were identified by the model as at risk for AKI. The patient was classified as at high risk of developing AKI (stage 2/3 KDIGO) episode if the risk-score exceeded the threshold. We computed the sensitivity of the model as the fraction of patients who developed AKI that were correctly identified by the model. The specificity was computed as the fraction of patients who never developed AKI and that were never identified at high risk by the model.

The performance values at the "knee point" of curves were chosen as the best performance values for each dataset. At this working point, i.e. at a specific risk-score threshold, we calculated the sensitivity, specificity and log likelihood ratio.

The *lead-time*, i.e. the number of hours between the first time the risk-score goes above the specific threshold and the AKI (stage 2/3 KDIGO) event, was also calculated for each patient.

### Statistical analyses

Data were reported as median and interquartile range or as absolute number and percentage, as appropriate. The accuracy of Random Forest Model for predicting the study outcome (i.e. AKI (stage 2/3 KDIGO) in the study populations was investigated by calculating the area under the receiver operating characteristic curve (auROC) as well as by assessing sensitivity and specificity. Positive and negative likelihood ratios (LR) were also calculated.

## Results

### Preparation of datasets

**Fig 1:**
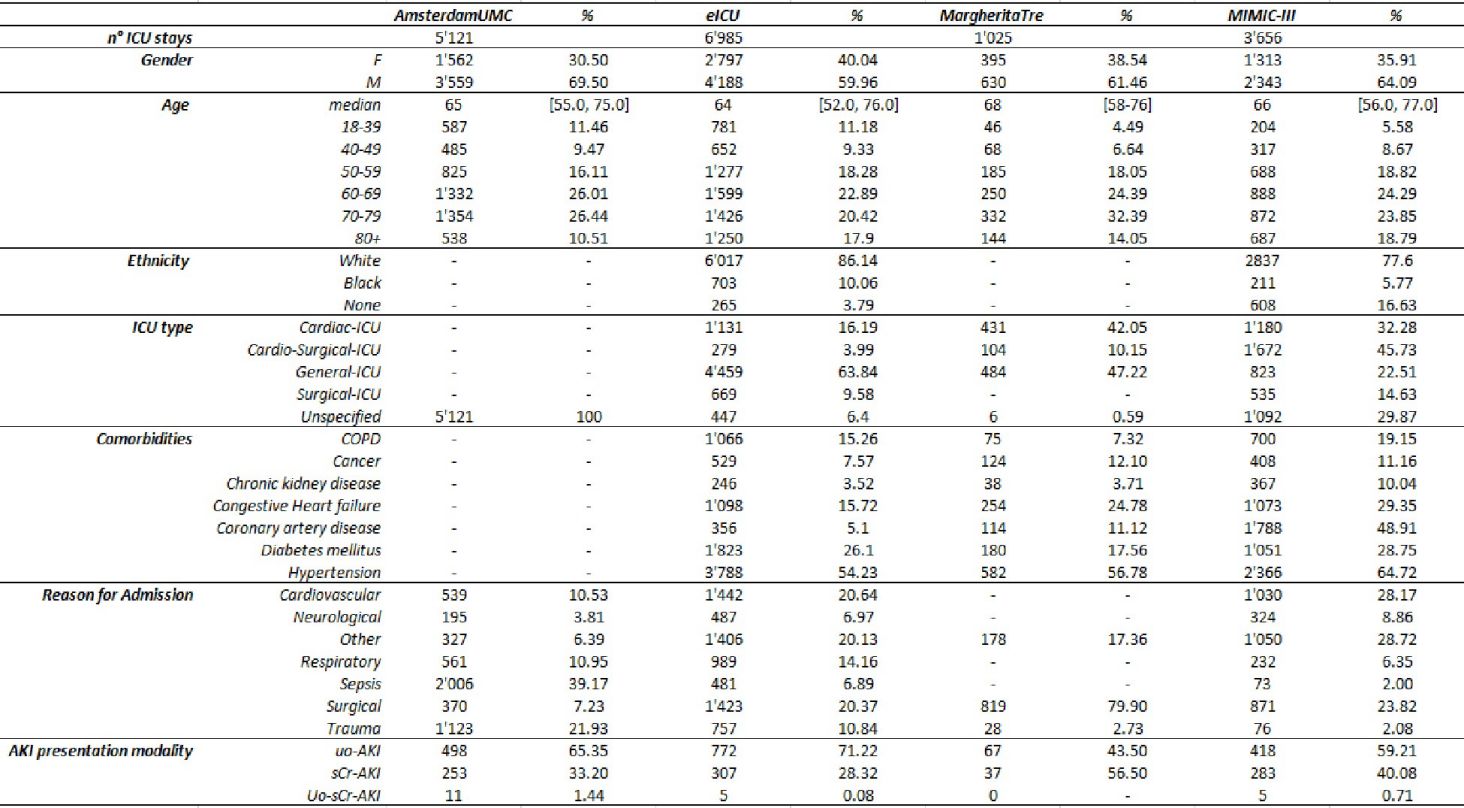
Main characteristics of the included population.

After applying the exclusion criteria and pre-processing steps, a total of 16’760 unique ICU admissions belonging to 16’454 patients were analyzed from patients of different regions (US, Europe) and multiple ICU centers (145). A total of 3’656 ICU admissions (from 3’592 unique patients) were obtained from the MIMIC-III dataset, 5’121 (from 5’011 unique patients) from the AmsterdamUMC dataset, 6’985 (from 6’835 unique patients) from the eICU dataset and 1’025 (from 1’016 unique patients) from the Margherita 3 dataset. The characteristics of populations are reported in Fig 1.

Datasets from different regions reported different distributions of ICU types. In the Netherland (Europe) patients are split in Medium or Intensive Care Units. In Italy, patients are mainly admitted into a General ICU or Cardio-Surgical ICU, while in the US more specific sub-types of ICU are present. In addition, a higher number of patients with chronic kidney disease (CKD) and coronary artery disease (CAD) as chronic comorbidities was registered in the US population than in the other ones. The comorbidities were identified through the use of ICD-9 and ICD-10 codes in those databases where the information was present.

Training of the algorithm was executed on 5’284 ICU admissions, representing the 60% of MIMIC-III and AmsterdamUMC populations. The remaining patients of MIMIC-III and AmsterdamUMC were allocated to the “calibration group” (1’744 ICU stays) to calibrate the predictive model, and to the “internal test group” (1’749 ICU stays) to evaluate the predictive model performances. Patients were randomly allocated to training, calibration and internal test groups.

**Fig 2:**
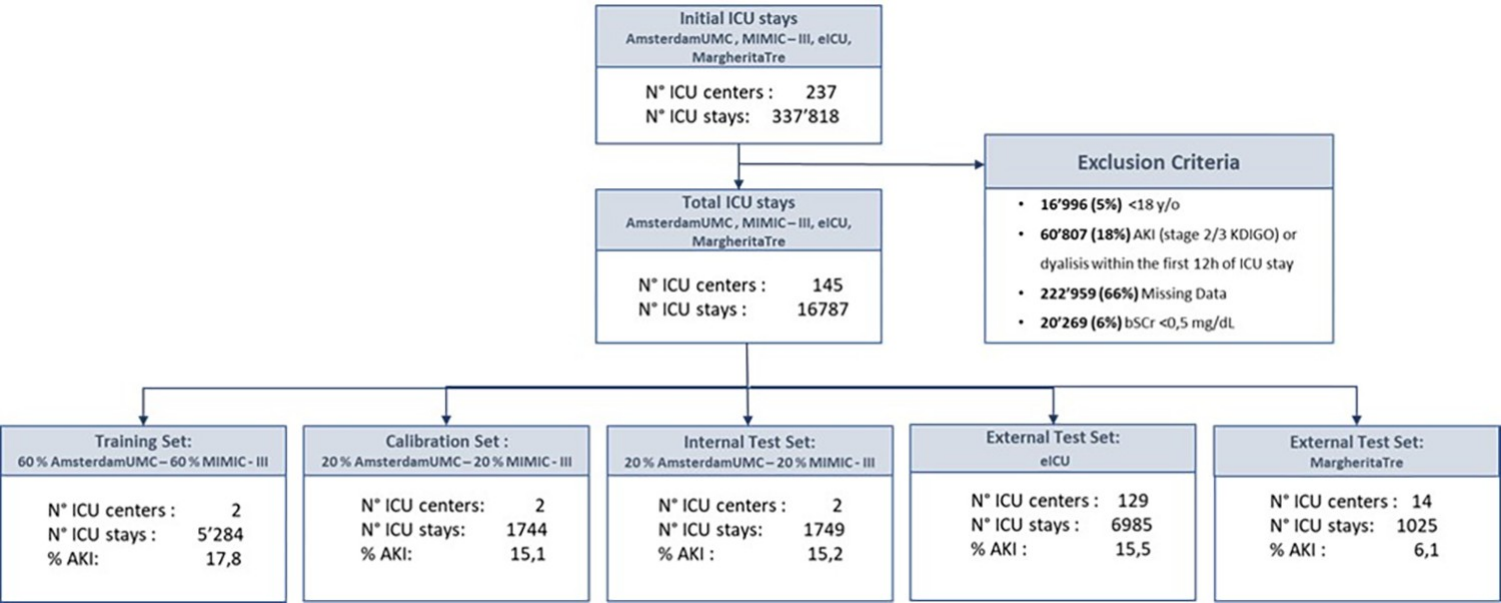
Data split into sets. N° ICU stays = Number of ICU stays associated with unique patients in each dataset.

The external validation of the model was executed on the two remaining independent multicenter databases, eICU and MargheritaTre, in order to prove the model performance and its ability to generalize on different geographical populations. This consisted of a total of 8’010 different ICU stays (Fig 2).

In the diverse patient cohorts, we identified an incidence of AKI (stage 2/3 KDIGO) varying in the range from 6% up to 18%. Among all episodes, AKI determined by Urine-Output criteria following KDIGO guidelines (Uo-AKI) was more frequent in almost all cohorts (> 55%) with respect to AKI determined by sCr (sCr-AKI). An exception was observed for the Italian dataset MargheritaTre, where only ~ 40% of AKI were Uo-AKI. In all cohorts the portion of males is higher than females and within the range 60–70%, the median ages are between 64 and 68. Main patient’s characteristics are summarized in Fig 1.

### Feature selection

**Fig 3:**
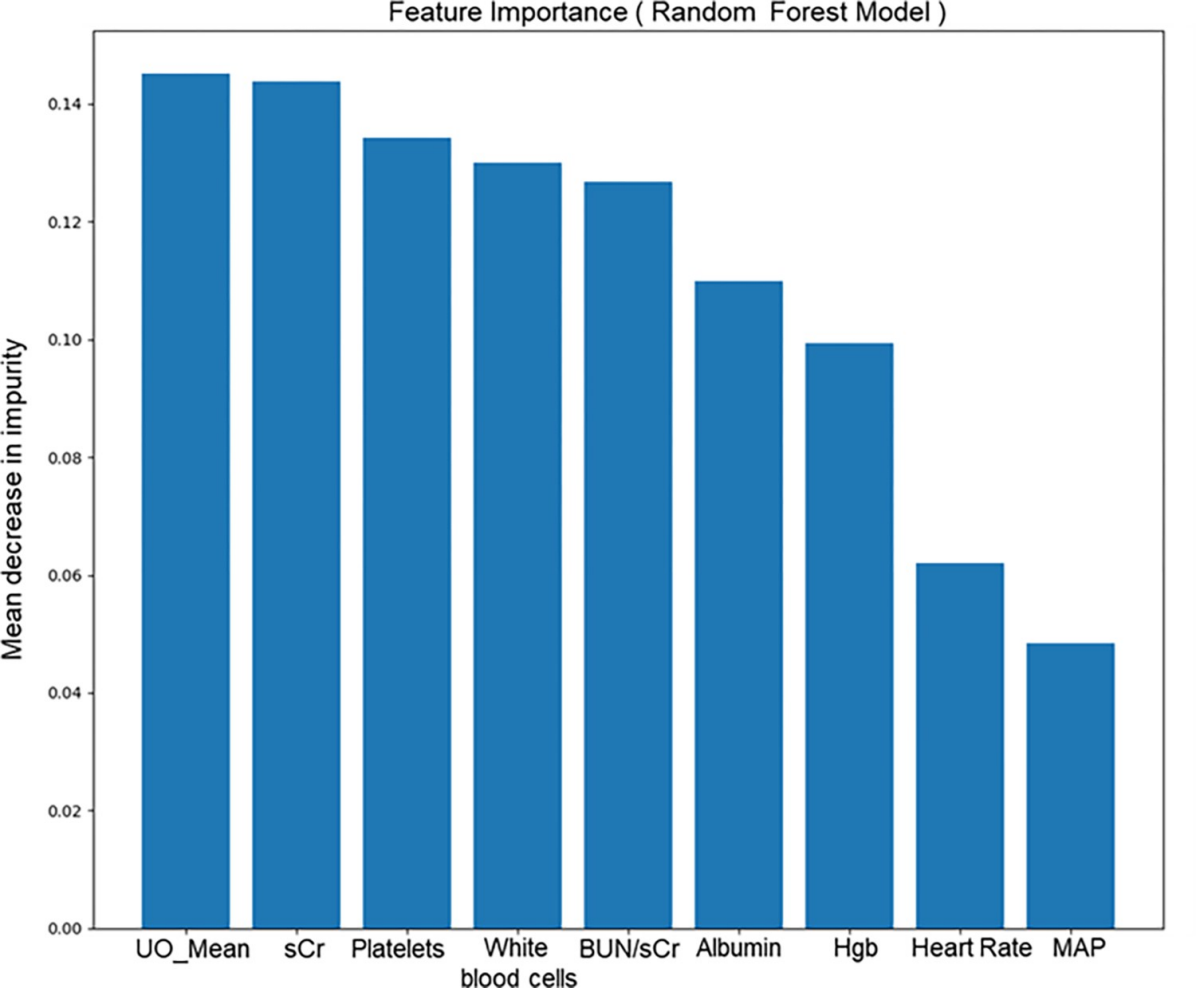
Feature importance for the Random Forest classifier. Each bar represents the contribution given from that variable in the classification process (Gini importance [21]).

To develop our predictive model for the early prediction of risk to develop AKI (Stage 2/3 KDIGO), we started the analysis by taking into account a series of clinical and demographic data, generally believed to have a role in AKI development. Subsequently, a feature selection was conducted based on the model’s feature importance to identify a narrow set of highly predictive parameters. The 60% (3’861 stays of which 18.7% AKI) of patients from MIMIC-III and AmsterdamUMC datasets were included in this step. Features were inserted one by one according to their Gini importance (Fig 3) as input features of the model and the auROC in predicting AKI (Stage 2/3 KDIGO) was calculated. Predictive performances of the model improved up to the inclusion of Urine Output, sCr and blood urea nitrogen, platelets, white blood cells, heart rate, MAP (Table 2). Nevertheless, the MAP was not included in the final model due to its low contribution to the predictive performances, as confirmed by an increase in auROC << 1% and its low availability in all analyzed datasets. Actually, less than 40% ICU patients reported MAP measurements.

**Table 2:** Results in terms of auROC obtained by adding new input parameters to the model.

*Clinical Parameters*	*auROC*
[‘UO_Mean’]	0.7971
[‘UO_Mean’, ’sCr’]	0.7688
[‘UO_Mean’, sCr’, ’platelets’]	0.7974
[‘UO_Mean’, ’sCr’, ’platelets’, ’white blood cells’]	0.8205
[‘UO_Mean’, ’sCr’, ’platelets’, ’white blood cells’, BUN/sCr’]	0.8336
[‘UO_Mean’, ’sCr’, ’platelets’, ’white blood cells’, BUN / sCr’,’albumin’]	0.8446
[‘UO_Mean’, ’sCr’, ’platelets’, ’white blood cells’, BUN / sCr’, ’albumin’, hemoglobin]	0.8476
[‘UO_Mean’, ’sCr’, ’platelets’, ’white blood cells’, BUN / sCr’, ’albumin’, hemoglobin,’heart rate’]	0.8569
[‘UO_Mean’, ’sCr’, ’platelets’, ’white blood cells’, BUN / sCr’, ’albumin’, hemoglobin, ’heart rate’, ’MAP’]	0.8604

Following the feature selection step, the final list of parameters included: the mean of 6 hours of hourly Urine Output normalized by Adjusted Body Weight of the patient (UO_mean), sCr, Platelets, BUN, White Blood Cells count, Hemoglobin, Albumin and Heart Rate. Patients used in the Feature Selection step were included in the final set used to train the model and constituted about 44% of the training sets.

### Model calibration

**Fig 4:**
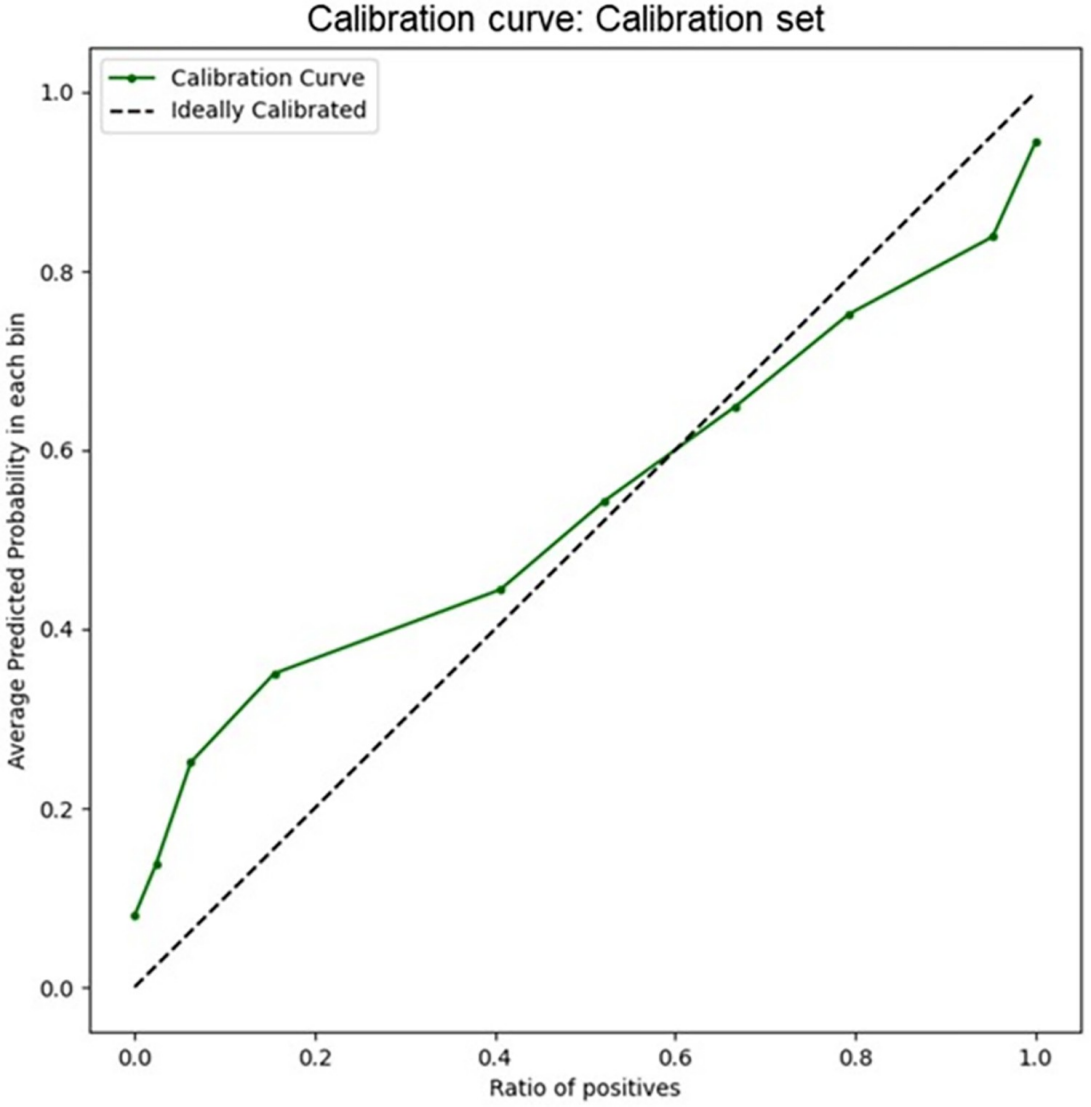
Calibration curve of the Random Forest model. Model predictions were grouped into 10 buckets. the average model risk prediction is plotted against the fraction of positive labels in that bucket. The dashed black-line represents the ideal calibration.

To ensure the trustworthiness of the returned risk probability predictions, we evaluated the calibration of the model when tested on patients from the Calibration set (belonging to MIMIC-III and AmsterdamUMC sets) by calculating the reliability plot. Model predictions were grouped into 10 buckets, with the average model risk prediction plotted against the fraction of positive labels in that bucket (Fig 4). We obtained a low Brier Score for the predictive model, equal to 0.094, indicating a well-calibrated classifier [22].

### Internal test

**Fig 5:**
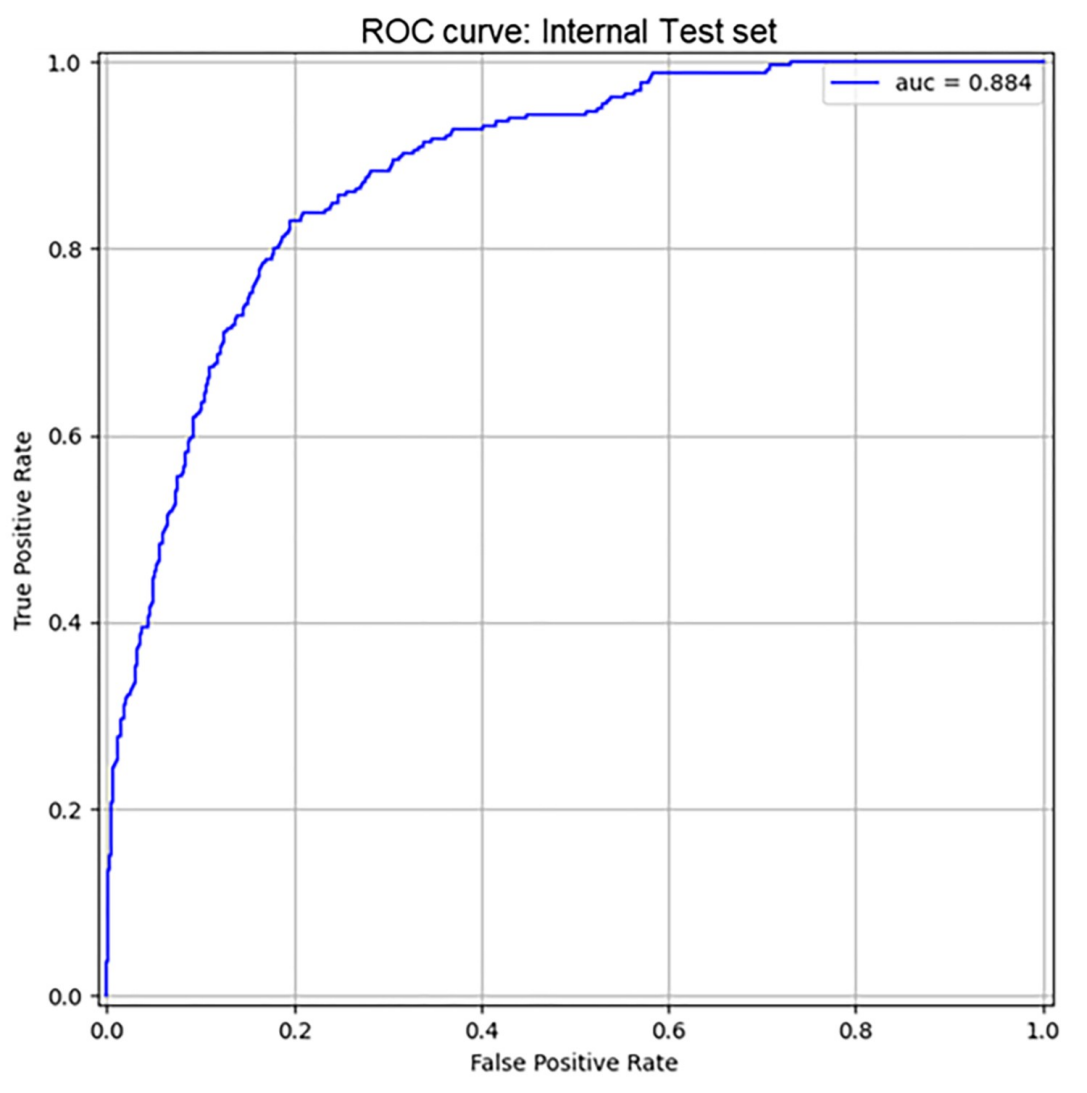
ROC curves of Random Forest model on internal test dataset. AUC = Area Under ROC Curve on prediction of diuresis and creatinine based AKI (stage 2/3 KDIGO).

The Random Forest model obtained an auROC of 0.884 [0.864–0.905] for AKI 2/3 prediction (Fig 5) when evaluated on the internal test set. At the sensitivity of 82%, the specificity was equal to 80% with a positive likelihood ratio (+LR) of 4.10 and a negative one (-LR) of 0.24 (Table 3).

**Table 3:** Results of the Random Forest model.

*Model*	*Dataset*	*Stays*	*% of AKI*	*auROC [iqr]*	*Sensitivity*	*Specificity*	*Median lead time (h) (AKI after the first 24h of ICU stay)*	*LR+*	*LR-*
***Random Forest***	Internal Test	1’749	15.2	0.884 [0.864–0.905]	82.0%	80.0%	15.5	4.10	0.24
***Random Forest***	eICU	6’985	15.5	0.877 [0.868–0.888]	83.0%	81.3%	14.0	4.44	0.23
***Random Forest***	Margherita Tre	1’025	6.1	0.911 [0.882.0.936]	82.5%	83.2%	23.0	4.91	0.20

auROC = area under receiving operator curve; LR + = likelihood positive ratio; LR- = likelihood negative ratio.

### External test

**Fig 6:**
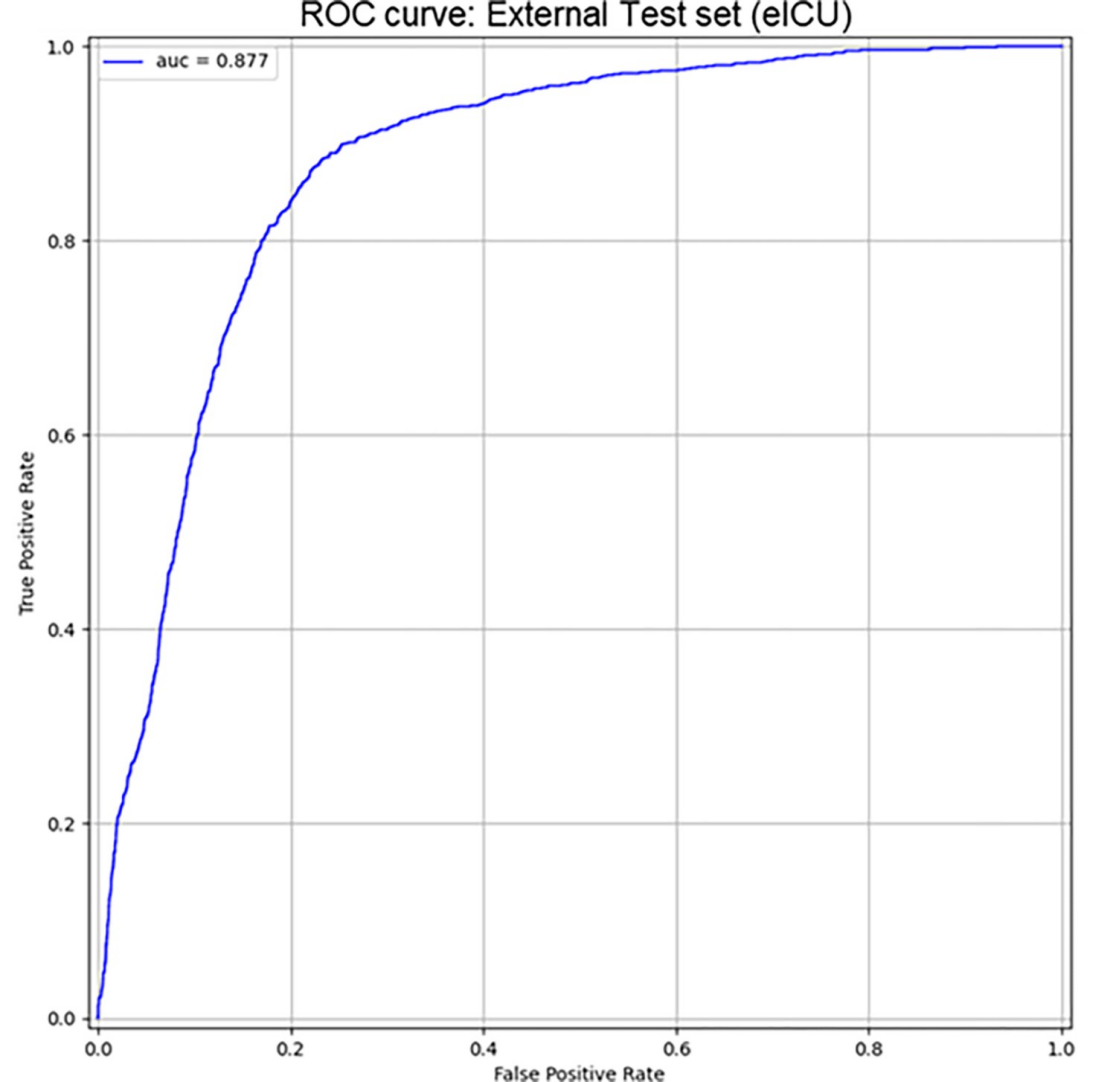
ROC curves of Random Forest model on eICU dataset. AUC = Area Under ROC Curve on prediction of diuresis and creatinine based AKI (stage 2/3 KDIGO).

On eICU population, the model achieved an auROC of 0.877 [0.868–0.888] (see Fig 6), with a specificity of 83% at the sensitivity of 81.3%. The corresponding values of positive (+LR) and negative (-LR) likelihood ratio were 4.44 and 0.23, respectively (Table 3).

**Fig 7:**
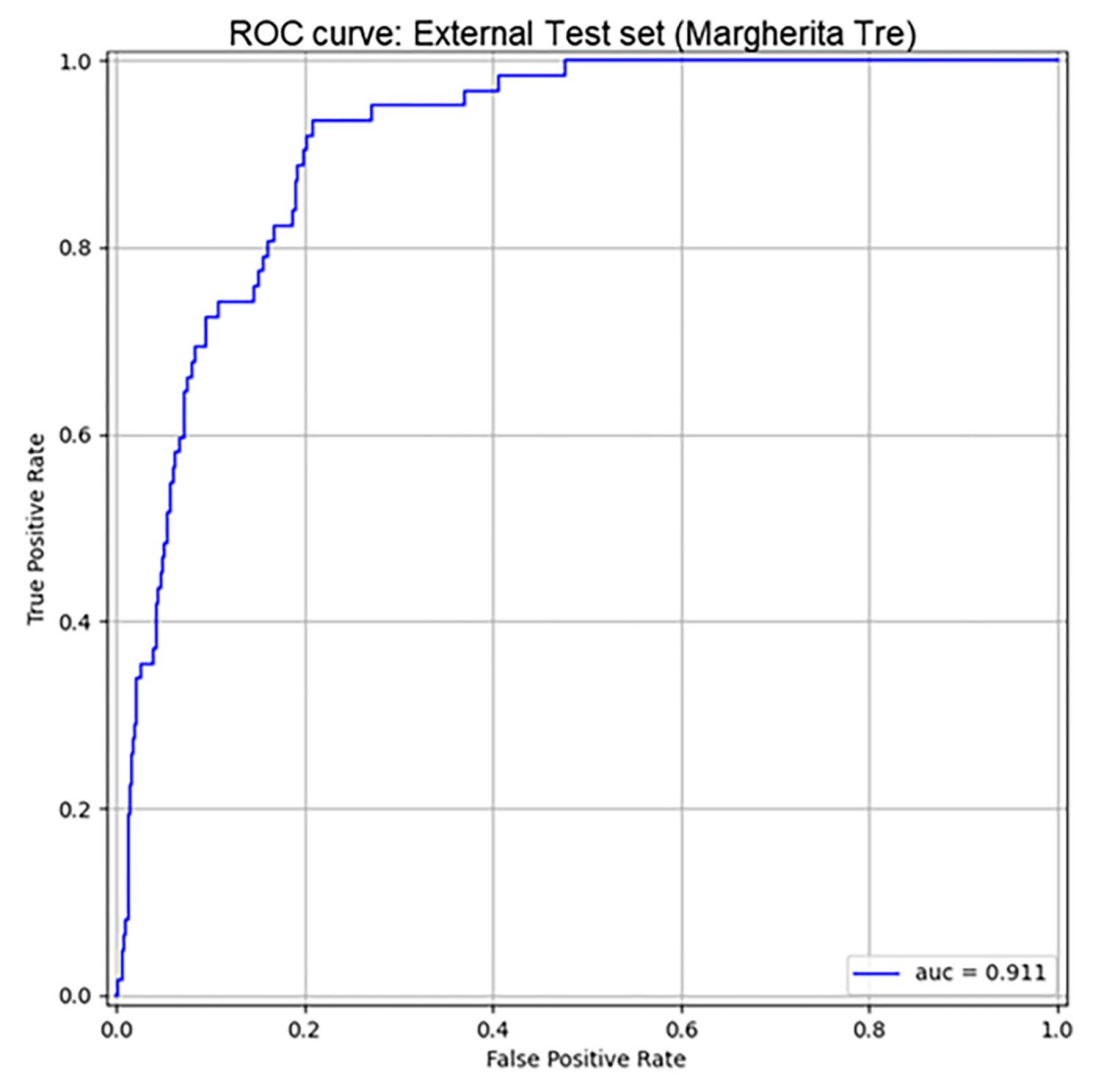
ROC curves of Random Forest model on MargheritaTre dataset. AUC = Area Under ROC Curve on prediction of diuresis and creatinine based AKI (stage 2/3 KDIGO).

The analysis on the Italian dataset Margherita Tre revealed that the model obtained an auROC of 0.911 [0.882, 0.936] and sensitivity of 82.5% at 83.20% of specificity (Fig 7). +LR and -LR were of 4.91 and 0.20, respectively (Table 3).

To compare the performances of our model with a simple baseline model, we conducted a further test by using the Logistic Regression model with the same input features and trained on the same training dataset: this resulted in lower predictive performances in terms of auROCs over all the test datasets (see Supplementary Info B, S2 Table in S1 File).

### Lead-time

The internal test showed a median lead-time of 9 h. We calculated the median *lead-time* when the AKI event occurred from 12^th^ to 24^th^ hours from ICU admission and after the first day of the ICU stay. The corresponding median lead-time were of 6 hours and 15.5 hours, respectively.

On the eICU external test, the resulting median *lead-time* for AKI episodes occurred in the first day of ICU stay was 8 hours, and 14 hours after the 24^th^ hour of ICU stay.

The obtained *lead-time* on MargheritaTre external test was 16 hours. Here, the same trend already observed for internal tests and on the eICU datasets was confirmed: the median *lead-time* within the first 24 hours was lower (8.5 h) than the lead time after the 1^st^ day of ICU hospitalization (23 h, see Table 4). This trend clearly shows how the developed model is able to dynamically improve itself by anticipating the prediction of the AKI event as soon as more data are available.

**Table 4:** Median lead time for AKI events occurred before and after24 hours from ICU admission.

*Data*	*N° IDS*	*% AKI*	*% AKI within 24h*	*Median lead time (h) (AKI within the first 24h of ICU stay)*	*Median lead time (h) (AKI after the first 24h of ICU stay)*
***Internal***	1’749	15.2	31.5	6.0	15.5
***External (eICU)***	6’985	15.5	34.8	7.0	14.0
***External (M3)***	1’025	6.1	24.0	8.5	23.0

**Fig 8:**
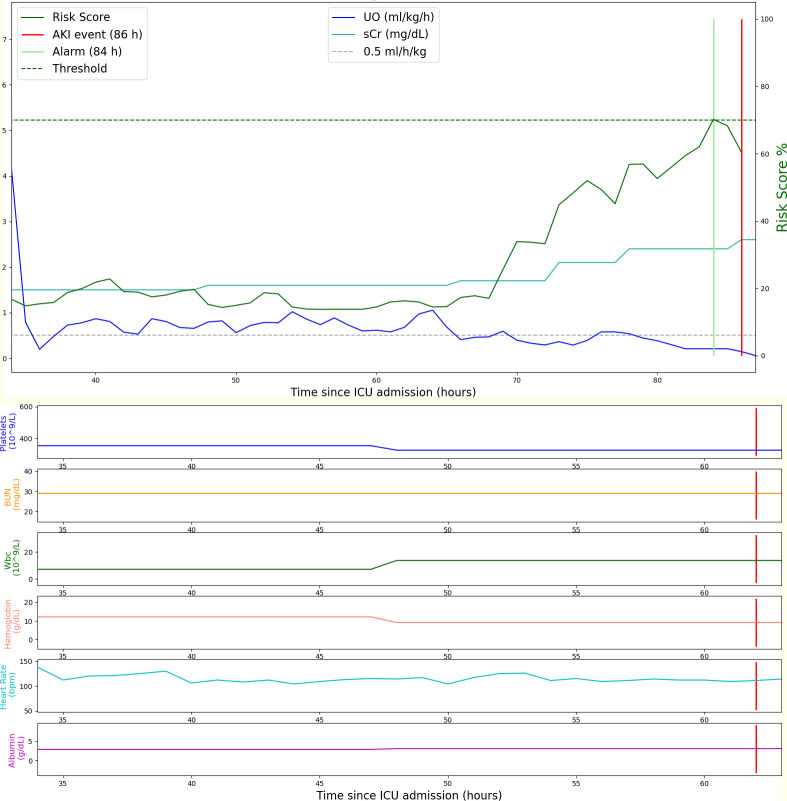
50 hours of ICU admission for a male patient aged 58 admitted for a pleural effusion in a medical ICU. (a) Urine output, serum creatinine and hourly risk score given by our predictive algorithm during the patient‘s stay. Serum creatinine increase determines the onset of AKI at h = 86^*th*^ (red vertical line). The alarm triggered by the model (green vertical line) is 16 hours before the event. (b) Laboratory variables trend.

An example of how our predictive model works as well as the associated lead-time is reported in Fig 8. A portion of a real ICU admission is presented. At every hour of ICU admission, the model provides a risk score in a percentage range from 0 to 100 related to the increased risk of developing AKI (stage 2/3 KDIGO) in the following hours. The model risk score exceeds the threshold at the 70^th^ h, which represents the moment when an alarm is fired by our model. Since the AKI 2 event occurs at the 86^th^ h of the ICU stay, the lead-time of the predictive model for this patient is 16 hours.

### Model performances in different ICU subpopulations

To evaluate the potential differences in predictive performances of our model when tested in diverse patient sub-populations, we measured the Average Odds Difference (AOD) metric in different patient cohorts. We considered free from bias a test result in accordance with acceptable values in the range: [-0.1, 0.1] [23]. All the analyses were both conducted on internal and external test sets with fixed threshold enabling a recall of 80%. We evaluated the AOD of patients’ cohorts possessing different attributes such as age and chronicity of illness, where present.

**Fig 9:**
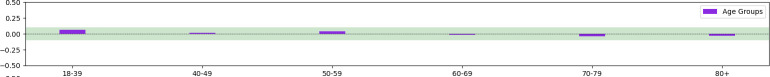
Bias Detection on internal test set. Fairness range [-0.1,0.1]. Bias are detected when bars exceede the green area.

**Fig 10:**
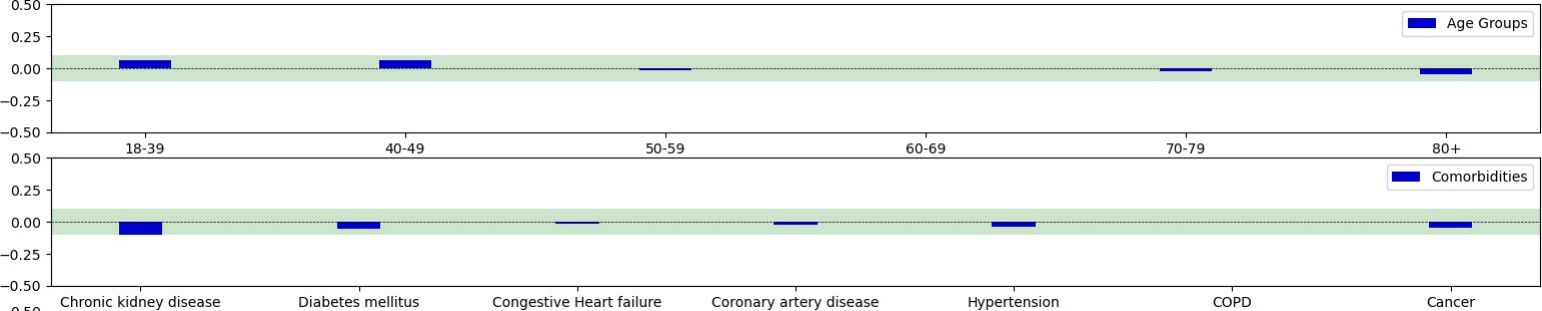
Bias detection on eICU dataset. Fairness range [-0.1,0.1]. Bias are detected when bars exceede the green area.

**Fig 11:**
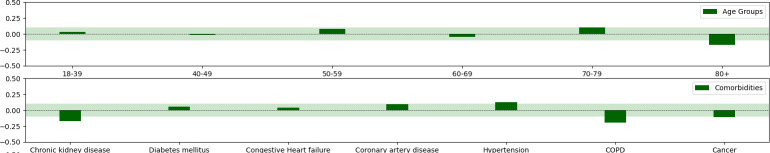
Bias detection on margherita Tre dataset. Fairness range [-0.1,0.1]. Bias are detected when bars exceede the green area.

Among all the parsed attributes, the model did not show any bias on Internal test and eICU datasets (Figs 9 and 10), while a very low bias was detected in the Margherita Tre patients. In particular, low biases were detected in the Chronic Kidney Disease, in COPD, and in 80+ age patient groups (Fig 11). Additional analysis can be found in Supplementary Info C, S3 Table in S1 File.

### Use of diuretics

To evaluate the impact of the use of diuretics during the ICU stay on the predictive performances of the model, we investigated the model behaviour on subgroups of patients that either received diuretics or not (Method described in Supplementary Info D in S1 File). The use of diuretics did not significantly impact the predictive performances of the model in both datasets. The observed impact can be attributed to the impact of the diuretic on the urine output parameter (Supplementary Info D, S1 and S2 Figs in S1 File).

### Model robustness to different bSCr estimation methodologies and to time-periods

As described in the Methods section, due to the absence of pre-ICU admission sCr measurements for most ICU patients, we used the nadir (minimum) in-hospital value of sCr as bSCr for the main analysis. To test the robustness of the developed predictive model to different methodologies of b-sCr estimation, we performed a sensibility study to evaluate predictive performances while varying the estimation methodology in the test datasets. An auROC higher than 0,88 was obtained in all datasets for all bSCr methodologies, confirming the robustness of the model when using different bSCr estimation (Method and Results described in Supplementary Info E, S4 Table in S1 File).

Moreover, to ensure that possible differences in patients’ phenotypes or in clinical practice over different time-periods did not impact negatively on the model predictive performances, an additional analysis was conducted by testing the model on samples of patients admitted in different years groups, ranging from 2003 up to 2022. Low changes in model performances (difference in auROC ~ 2%) was observed both for US data and the Netherlands data confirming the model’s reliability over time (Supplementary Info F, S5 Table in S1 File).

## Discussion

The changes of Acute Kidney Injury epidemiology highlight the necessity of awareness and care strategy modifications, as underlined by the International Society of Nephrology with the *0by25* initiative for AKI:”*zero preventable deaths by 2025”* [24]. Actually, a continuous increase of AKI incidence has been reported over the years [25–27]. Moreover, during the last two years, COVID-19 doubled the incidence of AKI in patients admitted to hospitals compared to non-COVID-19 ones [28]. Several studies report an incidence of AKI in COVID -19 hospital patients ranging from 30 to 50%, with a peak in Intensive Care Unit of about 70% [29–31].

In this context, the ability to predict the development of AKI in hospitalized patients could be an opportunity to adopt any strategy useful to avoid the event. The conceptual basis is reported in 0by25 initiative for AKI and it can be resumed in two points: the first, “AKI is potentially preventable and treatable with timely intervention”; the second is the possibility to act on “modifiable main risk factors” [24].

The present study represents the development of a prediction model for AKI stage 2 and 3 KDIGO in patients admitted to any ICU. The model is based on urine output, biochemical and haematologic data collected during ICU stay; it can predict AKI episodes occurring after the first 12 hours from admittance, requiring at least 6 hours of available data. We have analysed the predictive accuracy of mathematical models observing better performances with Random Forest instead of Logistic Regression models. The elevated accuracy of the algorithm, with a resulting auROC above 0.87 on all datasets, all patient subpopulations and ICU types, confirmed the general validity of AKI prediction model obtained with the Random Forest analysis. Moreover, after the first day in ICU, our model was able to trigger an alarm from 14 to 23 hours before the AKI event, remarkably improving its prediction performances with respect to the beginning of ICU admission.

Artificial Intelligence has been used in several applications of healthcare, as the prediction of an event like AKI. This emerging investigation field, not explored until the last 5–6 years, remains limited to few researchers: “artificial intelligence” AND “AKI” studies appear in PUBMED—year 2019 –just 6 times, while in year 2021 appear 33 times. Recently, the prediction was a “static” information derived from the analysis of data collected at the moment of Hospital or ICU admission, but now the analysis of “dynamic” data begins to be reported [32].

Other tests can be used to predict the development of AKI in intensive care unit. Clinical studies with biomarkers, such as TIMP-2 x IGFB 7, NGAL, or KIM-1, support their use in prevention and management of AKI, although the accuracy to predict AKI could change with the setting of patients, and substantial gaps in knowledge remain [33]. Concerning the accuracy to predict AKI, Pan and co-authors [33] analysed 110 studies which included 38725 patients: 21.5% of them developed AKI. The timing of biomarkers determination varied significantly between the study ranging from few hours from surgical intervention or ICU admittance to three days. NGAL/creatinine HSROC was 91.4%, KIM-1 = 84.4%, TIMP-2 x IGFB 7 cutoff 0.3 = 75.2%, and cutoff 2 = 78.8 [33]. The conclusion of the study is that biomarkers can permit an early identification of AKI before SCr changes, that is when the damage may be reversible. A limit of biomarkers can be linked to the heterogeneous sensitivity and specificity in different setting and patients, being not completely specific for acute kidney injury. Some of them can be elevated in the presence of sepsis or CKD [34], and it is necessary to investigate whether different etiologies of AKI affect the predictive accuracy of biomarkers [33].

The determination of some Biomarkers can be altered from the presence of proteinuria or albuminuria which can interfere with the detection [35].

A difference between the use of biomarkers and our study is essentially based on timing of AKI prediction. Our model predicts continuously the AKI development during the entire permanence in the intensive care area. Another important aspect is the feasibility of the proposed tool, which application does not require specialized laboratories or elevated costs. Through the use of AI techniques, our predictive model is able to exceed this limitation and provide a real-time prediction updated every hour, lifting physician from the responsibility of timely executing a diagnostic test. Another important advantage of our AI model is its automatic nature. For model operation, no additional work is required by the clinicians or nurses: the model uses only routinely collected data. The model described in this study have the potential of running in background and trigger an alarm when AKI-related trends are identified.

Thomasev and co-authors [19] approached AKI prediction with a continuous model based on sliding 6 hours interval record with a lead-time of event prediction corresponding to 48 hours. That model predicts 90.2% of AKI requiring dialysis with a lead-time up to 48 hrs and a ratio of two false alerts for every true alert. A limitation of the study was the low sensitivity, equal to 55.8%, meaning that half of the AKI episodes are missed, and the complexity of the model, a deep learning process which used more than 300 features. Two other aspects must be considered when analysing AI and AKI prediction: the first is the external validation, not frequently performed, and the second is the prospective analysis. Liang and co-authors [36] analysed data from MIMIC-III and the database of General Intensive Care Unit of Zhejiang University School of Medicine, the SHZJU-ICU. The Internal validation had an AUC of 0.86, confirmed with the AmsterdamUMC external validation. The main variables which demonstrated to be the most important parameters were sCr levels, urine output, blood urea nitrogen level, temperature and length of stay in ICU. The internal validation demonstrated a sensitivity from 83 to 84% and a specificity from 75 to 79%, not significantly different when external validation was considered. The time to event prediction was 48 hours. The prospective validation study on a limited number of AKI revealed a similar auROC, with a sensitivity of 72% and specificity of 80%.

Our predictive model overcomes several limitations that hindered the available predictive models to enter the routine clinical practice: first of all, the predictive performances. Indeed, auROC in both internal and external validations appear to be higher than previously published predictive models for AKI stage 2 and 3. Second, our model employs only a small number of routinely-collected predictive parameters. This is crucial to enable the implementation of the model in a real-world scenario. Moreover, the clinical variables which have a predictive role are also partially different than published literature, confirming the importance of creatinine and urine output, but also adding platelets, Blood Urea Nitrogen/sCr ratio, White Blood Cell, Haemoglobin, Albumin, Heart Rate. These results confirm the role of standard laboratory determination as blood count [31, 37–40] or albumin [41, 42] in the prediction of AKI. Although many clinical variables have been investigated in this study, evidence in the literature demonstrates that variables such as MAP may have a role in the early detection of reduced renal function. Therefore, the inclusion of this parameter in the model will be tested in forthcoming prospective validations [43].

Third, the predictive model was robust over different types of Intensive Care Units, either medical or post cardiac-surgery ICUs. Fourth, use of diuretics, that could introduce a bias in the performance of the predictive model, did not significantly reduced the performances: a post-hoc analysis revealed that AUC did not differ significantly between patients under diuretic treatment (auROC = 0.855) or not (auROC = 0.9). Fifth, our predictive model did not show any bias in different patient populations under analysis and it was well calibrated, as demonstrated by Brier score < 0.1. Eventually, our predictive model was also able to predict both AKI defined with Urine Output-based criteria and Creatinine-based criteria, differently from most published models that focused only on one of the two diagnostic criteria and AKI phenotypes [19, 44]. This is important because previous studies have shown that Creatinine-based AKI represent only a fraction of the total AKI episodes acquired in ICU [45].

In summary, if investigators need to demonstrate that prediction tools may have the potential to be applied in a real-world setting [46], our study is able to confirm it: the Random Forest model for AKI 2/3 prediction was externally validated and optimally calibrated, resulting applicable to different sets of critically ill patients.

The proactive identification of AKI obtained with this analytical model can allow e-alerts, which in essence can be thought as a clinical decision support system (CDSS) to help and improve health and healthcare decisions. We have observed a lead-time from e-alarm to clinical event that ranges from 8 to 15 hours. The question is linked to the time required for changes or implementation of diagnostic and therapeutic approaches able to preserve kidney from an impairment, and to the efficiency of such approach in terms of reversible-event reduction, in hospital stay decrease and mortality reduction.

The time-to-event 8 hours could be sufficient for adopting clinical strategies able to decrease AKI 2/3 KDIGO development, as could be in the case of reversible injuries.

E-alarms are expected to give on time information to medical staff to aid patient care, permitting early clinical investigations and preventive specific treatments. A review performed in 2017 from Lachance et al [47] failed to demonstrate the clinical utility of e-alarm, and similarly did Wilson [48], but the characteristic of these alarms were deeply different from those we can obtain with AI process. Previous studies were based on contemporary e-alarm production to AKI event, determined mainly on the base of sCr changes. Combining e-alarm to an AKI care bundle, a decrease of mortality and hospitalization days were already reported [49, 50]. We believe that the utility of an e-alarm driven from Artificial Intelligence methods could help clinicians, but we need to test properly this hypothesis. A suggestion about the utility of an integrated approach to early prediction of an event was recently published by the group of Iannello and co-authors [51]. The authors demonstrated an improvement in adjusted mortality of patients admitted to an emergency department with the use of an early warning sepsis system, capturing clinical and haematological parameters, and generating an automated decision support, integrated in the electronic health record system.

## Study limitations

Several limitations are present in our study, which derive from the nature of data sources and big data analysis. The first is the retrospective nature of the analysis, meaning that we necessitate to perform a prospective study to further validate model performances as performed by Liang [36]. Moreover, a prospective interventional study evaluating whether and how the availability of our AKI risk prediction score can affect clinical outcome is needed. The second limitation deals with the imputation of missing data, that may have introduced errors. To guarantee our model working properly, data need to be provided with an hourly frequency. With this scope, as previously mentioned, a forward filling methodology was used to impute missing data, as performed by several investigators [52–54].

To evaluate the size of data imputations, we conducted an analysis on the median acquisition rate of clinical parameters among the population eligible for the study. Those results (shown in Supplementary Info A, S1 Table in S1 File) are strictly correlated to the rate of missing values imputed. The 75^th^ percentile of each clinical values from laboratory tests appears approximately equal to one day, except for the albumin in MIMIC-III dataset, where measurements are the rarest. This underlines that our target population includes well-monitored patients. Despite we accepted missing values for a maximum period of 4 days, 75% of the laboratory data are in the majority of cases available with higher frequencies, introducing low classification bias. The third limitation is the estimation of a bSCr value. Various methods for determining the reference bSCr values were already described in the literature and there is no current standard methodology. The use of a nadir (minimum) in-hospital value, when a pre-hospital baseline is not available, was described by several authors [55, 56]. In our analysis, we used a nadir in-hospital value (rather than median or mean). Siew et al [55] found that the use of a nadir in-hospital sCr value led 81.7% of sensitivity and a specificity of 79.8% for the diagnosis of AKI, when compared with actual pre-hospital admission baseline values. However, few misclassifications were found approximately equals to 2.8% for AKI stage 2 or 3 KDIGO (the primary endpoint) and about 1.5% for the negative endpoint; a similar result was also reported by Závada et al [57]. Furthermore, Siew et al [55] did not use Urine Output criteria in their AKI stage calculation, and the incorporation of Urine Output criteria, as we did in our study, will tend to mitigate inaccuracies associated with the sCr reference value [57]. To evaluate its robustness, our supplementary analysis showed that the developed predictive model is robust to the different bSCr estimation methodologies.

The fourth limitation of our study could be the exclusion of patients with a bSCr below 0.5 mg/dL. Several considerations should be taken into account: it is known that SCr is related also to muscle-mass and ICU stay can be responsible of acute modifications as denutrition, reduction of muscle-mass, increased catabolism, sepsis, volume overload or de-hydratation [58]. Fluid overload can influence serum creatinine values, and the adjustment with fluid management could identify unrecognised AKI [59]. Intensive care patients may have a decreased production of creatinine as the consequence of muscle-mass reduction worsened by subclinical hepatic injury. Sepsis reduces energy production and metabolic rate, resulting in a possible decrease in muscle production of creatinine [60]. Variability can be due to false elevation of serum creatinine as interference with hyperglycemia, delayed centrifugation, hemolysis, high total proteins when used Jaffe assay. Another factor linked to acute effect on creatinine is the use of some drugs, as trimethoprim and cimetidine [58].

To reduce the possible bias, we arbitrarily adopted as exclusion criteria a cutoff for basal SCr at ICU admittance inferior to 0.5 mg/dL, and we have analysed only AKI stage 2 and 3 due to its larger increase in comparison to AKI stage 1.

## Conclusion

This study, based on a retrospective analysis of big data from ICU patients, demonstrates that Artificial Intelligence, in particular the use of a Random Forest model, could help clinicians in predicting the risk of AKI development. Our study gives additional information to those present in the literature, in terms of a general prediction tool over a wide range of ICU patients, and regarding the precision of prediction, as confirmed by excellent calibration and external multi-centric validation. In particular, our model dynamically improves its performances after the first 24 hours of ICU admission, increasing the predictive lead-time. As the critically conditions of the patient get less unstable and the availability of data becomes higher, our model have the potential to boost its lead-time while guaranteeing the same accuracy. This can be translated into an increased time-window for clinicians to act and potentially prevent AKI. In summary, our study shows that our model could predict, with many hours in advance, patients at high risk of developing AKI (AKI 2/3 KDIGO). Our model uses only measurements routinely collected in the EHR. Due to the limitations of retrospective analysis, that is the heterogeneity or incompleteness of available data and the risk of possible biases, it is necessary to confirm our observations through a well-designed prospective study. However, the characteristics of the model and the high performance we have demonstrated using a large and heterogeneous cohort (multi-centric and multi-national) of ICU patients, overcoming available predictive models, indicates that the model implementation in the real-world has the potential to enable proactive management and prevention of AKI episodes in ICU patients. A prospective clinical trial to validate the performances of the model in real-world scenarios is currently under development.

## Supporting information

S1 File (DOCX)

## Abbreviations

AKI: Acute Kidney Injury
auROC: Area Under the Receiver Operating Characteristic Curve
bSCr: Basal value of sCr
DL: Deep Learning
EHR: Electronic Health Record
ICU: Intensive Care Unit
KDIGO: Kidney Disease: Improving Global Outcomes
ML: Machine Learning
sCr: Serum Creatinine
sCr: AKI Acute Kidney Injury Detected by Applying Serum Creatinine Criteria
Uo: Urine Output
Uo-AKI: Acute Kidney Injury Detected by Applying Urine Output Criteria
