# Supplementary material for: Continuous and early prediction of future moderate and severe Acute Kidney Injury in critically ill patients: Development and multi-centric, multi-national external validation of a machine-learning model
Source: PLoS One. 2023 Jul 25;18(7):e0287398. doi: 10.1371/journal.pone.0287398 (PMC10368244; doi:10.1371/journal.pone.0287398)
Supplement: S1 File — (DOCX) [file pone.0287398.s001.docx]

# Continuous and early prediction of moderate and severe AKI in critically ill patients: development and multi-centric external validation of a machine-learning model

## SUPPLEMENTARY INFO

### Acquisition Frequency of input parameters

The original average acquisition frequency of parameters in the different datasets is shown in S1 Table.

*S1 Table: Median acquisition frequency of parameters in the analyzed population.*

| ***Dataset*** | ***AmsterdamUMC*** | | ***MIMIC-III*** | | ***eICU*** | | ***MargheritaTre*** | |
| --- | --- | --- | --- | --- | --- | --- | --- | --- |
|  | *median(h)* | *IQR* | *median(h)* | *IQR* | *median(h)* | *IQR* | *median(h)* | *IQR* |
| ***Albumin*** | 21.44 | [14.4, 25.82] | 26.05 | [16.59, 50.42] | 19.79 | [10.98, 24.71] | 9.90 | [2.75,18.0] |
| ***BUN*** | 17.38 | [11.76, 24.22] | 15.17 | [11.85, 19.23] | 15.68 | [9.98, 22.37] | 8.70 | [6.19,13.62] |
| ***Heart rate*** | 0.97 | [0.02, 0.99] | 0.85 | [0.74, 0.95] | 0.84 | [0.61, 0.97] | 0.45 | [0.33,0.51] |
| ***Hemoglobin*** | 2.88 | [2.07, 4.78] | 11.83 | [6.76, 17.88] | 15.24 | [8.43, 23.06] | 10.80 | [7.74,17.26] |
| ***Platelets*** | 12.40 | [6.09, 18.76] | 14.83 | [9.78, 19.87] | 17.62 | [10.98, 23.67] | 10.80 | [7.73,17.26] |
| ***Serum Creatinine*** | 15.33 | [11.08, 19.62] | 15.11 | [11.79, 19.16] | 15.67 | [9.99, 22.32] | 17.00 | [13.37,23.59] |
| ***Urine Output*** | 1.33 | [1.18, 1.55] | 1.13 | [1.03, 1.35] | 1.47 | [1.08, 2.08] | 1.00 | [0.99,1.04] |
| ***White Blood Cells*** | 15.03 | [9.21, 20.26] | 15.77 | [10.98, 20.93] | 17.83 | [11.21, 23.68] | 10.80 | [7.73,17.26] |

### Baseline model. Logistic Regression

A 60% of patients from AmsterdamUMC and Mimic-III were used. The obtained results were lower than Random Forrest in terms of auROCs over all the test datasets.

S2 Table: Results of the Logistic Regression Model over the testing population

| ***Model*** | ***Dataset*** | ***ICU stays*** | ***% AKI***  ***(stage 2/3 KDIGO)*** | ***auROC [iqr]*** | ***median lead-time (h)*** |
| --- | --- | --- | --- | --- | --- |
| Logistic Regression | Internal Test | 1’749 | 15.0 | 0.811  [0.784 - 0.841] | 15.0 |
| Logistic Regression | eICU | 6’985 | 15.5 | 0.804  [0.791 - 0.818] | 14.0 |
| Logistic Regression | Margherita Tre | 1’025 | 6.1 | 0.870  [0.827 - 0.907] | 20.0 |

### Model performances on subgroups of patients from different ICU types

The analyzed population includes patients from various ICU types. For each ICU type we evaluated the model’s performance with the aim of demonstrating its applicability in all ICUs. The model exhibits high auROC values across most diagnostic groups, with changes lower than 10%.

S3 Table: Results over different ICU of admittance in testing population

| ***Model*** | ***Dataset*** | ***ICU type*** | ***% AKI***  ***(stage 2/3 KDIGO)*** | ***auROC*** |
| --- | --- | --- | --- | --- |
| ***Random Forest*** | External Test:  eICU | Medical | 16.46 | 0.915 |
|  |  | Surgical | 16.31 | 0.876 |
|  |  | Cardiosurgical | 10.75 | 0.928 |
|  |  | Cardiac | 14.94 | 0.865 |
|  |  | Neurosurgical | 10.74 | 0.866 |
| ***Random Forest*** | External Test:  Margherita Tre | Medical | 9.92 | 0.871 |
|  |  | Cardiosurgical | 1.92 | 0.966 |
|  |  | Cardiac | 2.78 | 0.956 |

### Impact of the use of diuretics during ICU admission on model predictive performances receiving diuretics

Access the impact diuretics use on the predictive performances of the model. We investigated the model behaviour in a subgroup of patients who received diuretics ( eICU: 1’707 ICU stays with AKI incidence of 13.65%, Margherita Tre: 477 ICU stays with AKI incidence of 7.76%) and the one composed of patients who did not received diuretics (eICU: 5’278 ICU stays with AKI incidence of 16.12%, Margherita Tre: 584 ICU stays with AKI incidence of 4.56%).


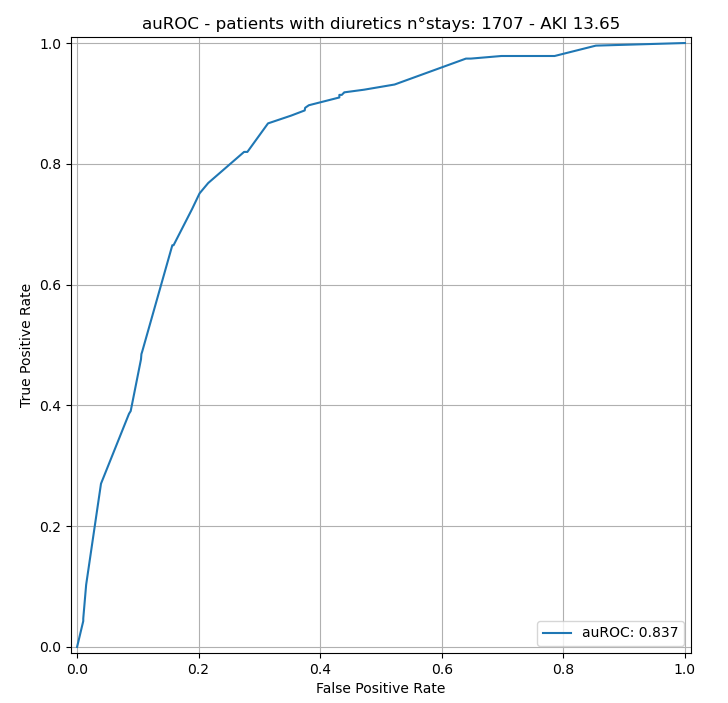

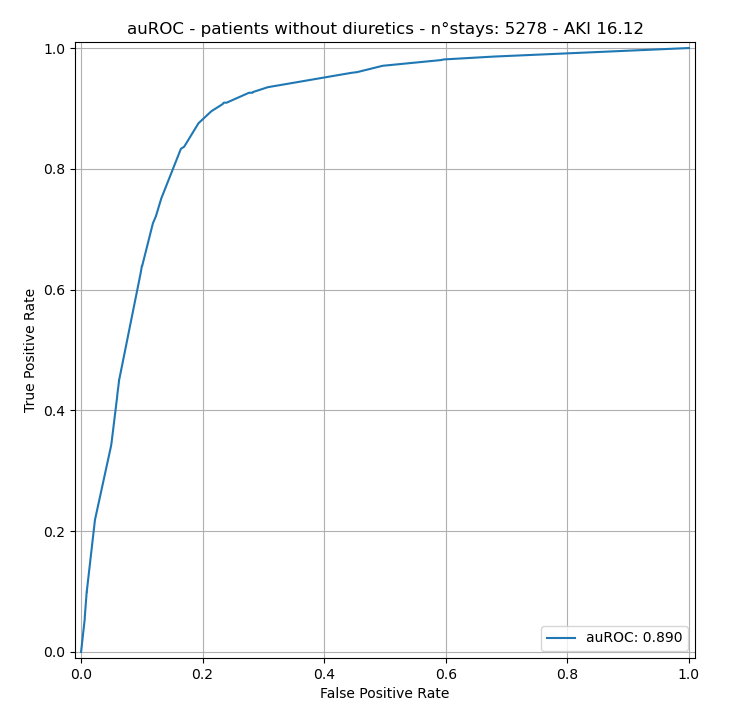


a)

b)

S1 Figure: Performance of Random Forest model tested on eICU dataset. a) Results on the subgroup of patients that receives diuretics. b) Results on the subgroup of patients that did not receive diuretics.


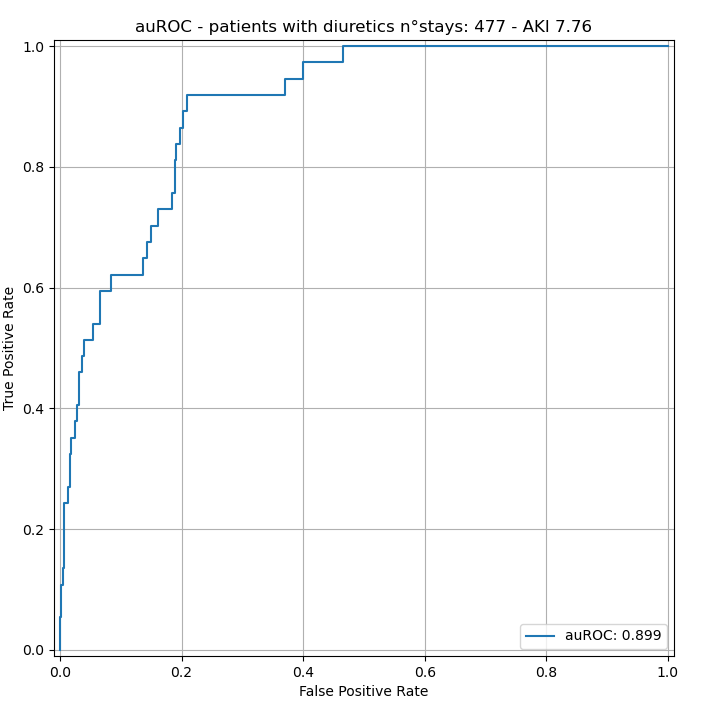

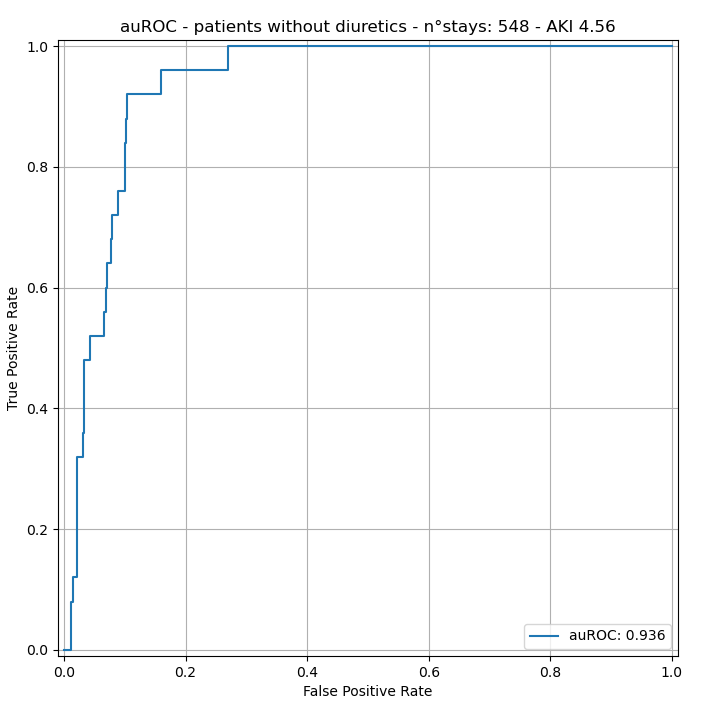


b)

a)

S2 Figure: Performance of Random Forest model tested on MargheritaTre dataset. a) Results on the subgroup of patients that receives diuretics. b) Results on the subgroup of patients that did not receive diuretics.

### Robustness of the model to different methodologies of creatinine baseline estimation

The AKI incidence in ICU settings derived by using KDIGO classification criteria is affected by the bias and limited accuracy of methods to estimate baseline creatinine.

To ensure the robustness and reliability of the model’s output, we evaluate the performances of different estimations of creatinine baseline summarized as follows:

- bSCr_1 (Nadir): the lowest sCr value of creatinine available in the database
- bSCr_2 (MDRD): sCr value derived by inverting the MDRD formula, with an eGFR of 75 mL/min/1.73 m2
- bSCr_3 (https://www.ncbi.nlm.nih.gov/pmc/articles/PMC4209880/):
  - 1. Median of all sCr values in the time range from six months to six days prior to ICU admittance;​
    2. if 1 unavailable, nadir value in the time range starting six days prior to ICU admittance and ending at ICU entrance, if available;​
    3. if 1 and 2 unavailable, value at the ICU admission​
- bSCr_4: the first sCr value available at the hospital admission​

S4 Table: Results using different creatinine baseline estimations in testing population

| ***Dataset*** | ***bSCr*** | ***bSCr***  ***median (iqr)*** | ***auROC*** |
| --- | --- | --- | --- |
| eICU | bSCr_1 | 0,79 [0,60 - 1,09] | 0,877 |
| eICU | bSCr_2 | 1,01 [0,82 - 1,07] | 0,883 |
| eICU | bSCr_3 | 0,88 [0,66-1,24] | 0,887 |
| eICU | bSCr_4 | 1,01[0,78 - 1,51] | 0,887 |
| Mimic-III | bSCr_1 | 0,70 [0,50 - 1,00] | 0,852 |
| Mimic-III | bSCr_2 | 1,01 [0,82 -1,07] | 0,893 |
| Mimic-III | bSCr_3 | 0,80 [0,60-1,10] | 0,879 |
| Mimic-III | bSCr_4 | 1,00 [0,80 -1,50] | 0,895 |
| AmsterdamUMC | bSCr_1 | 0,80 [0,61 - 1,04] | 0,908 |
| AmsterdamUMC | bSCr_2 | 1,03 [0,84 - 1,05] | 0,91 |
| AmsterdamUMC | bSCr_3 | 0,85 [0,67-1,08] | 0,914 |
| AmsterdamUMC | bSCr_4 | 0,97 [0,78 - 1,24] | 0,917 |
| Margherita Tre | bSCr_1 | 0,73 [0,64 - 0,88] | 0,911 |
| Margherita Tre | bSCr_2 | 1,02 [0,816 - 1,06] | 0,884 |
| Margherita Tre | bSCr_3 | 0,80 [0,60 -1,27] | 0,912 |
| Margherita Tre | bSCr_4 | 0,85 [0,67 - 1,18] | 0,908 |

In all the previously conducted analyses the estimation method “bSCr_1” was used for creatinine baseline. Table S2 shows the results obtained at varying bSCr estimates for the testing population. From the auROC point of view, we can observe a low percentage change up to +- 4% letting us to conclude that the model keeps its good predictive capabilities with minor to no fluctuations in lead-time except for Margherita Tre where, using bSCr methods 3 and 4, the alarm is triggered in advance. This may be attributed to a late AKI identification given a higher baseline.

### F. Model performances on subgroups of patients admitted in different years

S5 Table: Results over different years in testing population

| ***Admisson Year Group*** | ***Dataset*** | ***% ICU stays*** | ***% AKI***  ***(stage 2/3 KDIGO)*** | ***auROC*** |
| --- | --- | --- | --- | --- |
| 2003-2009 | AmsterdamUMC | 44,4 | 9,9 | 0,914 |
| 2010-2016 |  | 55,6 | 10,4 | 0,903 |
| 2016-2019 | MargheritaTre | 59,4 | 4,3 | 0,894 |
| 2020-2022 |  | 40,6 | 4,6 | 0,929 |
